# Management of Inoculation with *Bradyrhizobium japonicum* and Application of Vitamins for Hydroponic Soybean Cultivation

**DOI:** 10.1155/2024/4463693

**Published:** 2024-03-31

**Authors:** Alexandre H. de F. Lima, Josiane S. Salles, Eduardo P. Vendruscolo, Cássio de C. Seron, Rogério S. de Freitas, Sebastião F. de Lima, Gabriela R. Sant'Ana, Edilson Costa

**Affiliations:** ^1^State University of Mato Grosso do Sul, Cassilândia University Unit, Cassilândia 79540-000, Brazil; ^2^Instituto Agronômico de Campinas, Centro de Seringueira, Votuporanga 15505-970, Brazil; ^3^Federal University of Mato Grosso do Sul, Chapadão do Sul Campus, Chapadão do Sul 79560-000, Brazil

## Abstract

The exchange of technologies used in field cultivation for hydroponic systems can potentially increase plant development and grain production, requiring studies to verify the best management forms, such as growth-promoting bacteria and biostimulant compounds. With this in mind, the study aimed to evaluate the effect of the application of thiamine and niacin, alone and combined, to soybean plants in the absence and presence of inoculation with *B. japonicum* on the agronomic and physiological characteristics of the crop grown in an ebb and flow hydroponic system. Eight treatments were evaluated using *t*-test (LSD) and Tukey's test, both at 5% probability (*P* < 0.05), in addition to Pearson correlation and canonical variables. The treatments consist of inoculation with *B. japonicum* at 1 mL 500 g^−1^ seeds (with and without) and foliar application of four solutions (water, niacin (0.1 g·L^−1^), thiamine (0.1 g·L^−1^), and niacin + thiamine (0.05 g·L^−1^ + 0.05 g·L^−1^)). We found that inoculation significantly improved the parameters evaluated and resulted in a gain of approximately 84.8% in yield when compared by *t*-test (*P* < 0.05). In addition, the action of the vitamins was more significant when they were applied without the presence of *B. japonicum*, especially niacin, either alone or combined with thiamine, which increased yield parameters in this condition, identified when the Tukey's test (*P* < 0.05) was applied. We conclude that inoculation with *Bradyrhizobium japonicum* in soybean seeds grown in a hydroponic system significantly benefits the development and grain yield, mainly when combined with vitamin solutions. Niacin also has the potential to be used alone or combined with thiamine in noninoculated or inoculated hydroponic soybean crops, respectively.

## 1. Introduction

Although widely grown in open field conditions, there is growing interest in the production of soybeans in controlled environments to obtain specific products, such as the production of green beans, considered horticultural products. In addition, this kind of cultivation can be explored for specific conditions, such as for the establishment of protocols for cultivation in bio-regenerative life support systems [1]. Regardless of the purpose of the production, hydroponic cultivation can be used to produce healthy, more productive plants with low pesticide input, making it possible to use problem areas for conventional agriculture [2, 3].

Hydroponic systems also enable the efficient use of a series of technologies to obtain production increases, such as biostimulant products such as vitamins [4] and bacteria [5, 6]. In this sense, there is a migration of these technologies, used on a large scale under field conditions, to hydroponic cultivation. However, this technological introduction requires in-depth studies to identify the interactions with the cultivated species and make it possible to define more efficient management forms.

Inoculating seeds with *Bradyrhizobium japonicum* have several benefits for soybean crops grown under field conditions. In addition to fixing atmospheric nitrogen, the association between plants and bacteria results in an increase in hormone levels and greater absorption of other nutrients, such as phosphorus [7] and potassium [8], resulting in increased growth and grain yield. For hydroponic systems, the results obtained with the use of bacteria are contrasting, with indifferent responses to their presence [9], as well as positive responses in terms of nutrient absorption capacity, increased growth, and increased grain quantity and quality [10, 11].

As a complement to inoculation, among the various emerging technologies used to obtain better conditions for plant growth and production, B vitamins have been successfully applied both in field conditions [12] and in hydroponic cultivation [4]. Among these vitamins, thiamine (vitamin B1) acts as an enzymatic cofactor in glucose metabolic pathways, as a coenzyme in the decarboxylation-oxidation of pyruvate in the pentose phosphate and tricarboxylic cycle pathways (Krebs cycle) [13, 14]. Also, niacin (vitamin B3) is a substituted derivative of pyridine, and its active forms are nicotinamide adenine dinucleotide (NAD^+^) and nicotinamide adenine dinucleotide phosphate (NADP^+^). It acts in the transfer of hydrogen ions in energy-releasing reactions, as it is involved in many enzymatic oxidations and reduction reactions such as NADH and NADPH, as well as favoring the plant's defense system [15].

Generally, vitamins belonging to the B complex are constituent agents of coenzymes that act in catabolic reactions of macronutrients in the energy production process [14]. Some studies have shown positive effects on plant development, especially in conditions where stress is overcome, such as for rice [16], bean [17], and cucumber [18] crops, as well as in hydroponic systems, such as lettuce [4].

With the hypothesis that inoculation with *B. japonicum* combined with the application of B vitamins provides better conditions for growing hydroponic soybeans, the study aimed to evaluate the effect of the application of thiamine and niacin, alone and in combination on soybean plants in the absence and presence of inoculation with *B. japonicum* on the agronomic and physiological characteristics of the crop, grown in an ebb and flow hydroponic system.

## 2. Materials and Methods

### 2.1. Location and Characterization of the Experimental Area

The experiment was conducted in a protected environment at the State University of Mato Grosso do Sul (UEMS), in the Unit of Cassilândia (UUC), located in Cassilândia (19°07′21″ S, 51°43′15″ W, and altitude of 516 m). According to the Köppen climate classification, the region has a rainy tropical climate (Aw type) with rainy summers and dry winters.

The growing environment consisted of an air-conditioned greenhouse measuring 14.64 m × 6.40 m × 3.5 m (93.70 m^2^) + an anteroom measuring 3.66 m × 3.20 m (11.71 m^2^), with a total area of 105.41 m^2^. The roof and sides of the greenhouse were covered with 150-micron, light-diffusing, double-layer low-density polyethylene (LDPE) film with a 1.2 m × 0.15 m Humil Cool (CELDEX®) pad/fan climate control system. ALUMINET® 35% (“I”) aluminized thermo-reflective shading screen, movable, under the polyethylene film, which remained closed. With six internal metal benches (tables) measuring 1.10 m wide × 5.00 m long × 0.80 m high on a concrete floor. They also had a 0.35 m high concrete wall around the perimeter of the module and a 0.80 m wide concrete sidewalk around the module.

### 2.2. Experimental Design

The experimental design was randomized blocks in a 2 × 4 factorial scheme, with five replications, defined by the absence and presence of inoculation with *B. japonicum* and the application of four solutions via foliar spray. Plots consisted of one pot with two plants.

The eight treatments consisted of T1 = no inoculation + spraying with water, T2 = no inoculation + Niacin (0.1 g·L^−1^), T3 = no inoculation + thiamine (0.1 g·L^−1^), T4 = no inoculation + thiamine (0.05 g·L^−1^) + niacin (0.05 g·L^−1^), T5 = *B. japonicum* (1 mL 500 g^−1^) + spraying with water, T6 = *B. japonicum* (1 mL 500 g^−1^) + niacin (0.1 g·L^−1^), T7 = *B. japonicum* (1 mL 500 g^−1^) + thiamine (0.1 g·L^−1^), and T8 = *B. japonicum* (1 mL 500 g^−1^) + thiamine (0.05 g·L^−1^) + niacin (0.05 g·L^−1^).

### 2.3. Conducting the Experiment

The cultivar used, DM 75I74 RSF IPRO is medium-sized with an average cycle of 110 to 115 days, a 1000-seed weight of 156 g, moderate resistance to lodging, indeterminate growth habit with low branching potential, highly demanding on fertility, maturity group: 7.5. It has a light brown hilum with white flowers and gray pubescence, a cultivar with high yield potential and resistance to stem canker, bacterial pustule, and cyst nematode (race 3), as described by the manufacturer Don Mario seeds.

The application was made directly onto the seeds for the inoculation treatments, with 1 mL of inoculant (Total Nitro Max®) per 500 g of seeds. The inoculant used (Total Nitro Max®) is based on the bacterium *Bradyrhizobium japonicum*, with two strains recommended for Brazil, Semia 5079 and Semia 5080, developed with high-quality material, with 5 × 10^9^ CFU·ml^−1^, recommended for soybean cultivation, according to the manufacturer's information.

Black polyethylene pots with a volume of 0.5 L were used for sowing and supporting the soybean plants. The pots were filled with the commercial substrate Carolina soil®, made up of *Sphagnum* peat, expanded vermiculite, dolomitic limestone, agricultural gypsum, and NPK fertilizer, with a pH of 5.5 ± 0.5, electrical conductivity (EC) of 0.7 ± 0.3, density of 145 kg m³, water retention capacity (WRC) of 55%, and maximum humidity of 50%.

**Figure 1:**
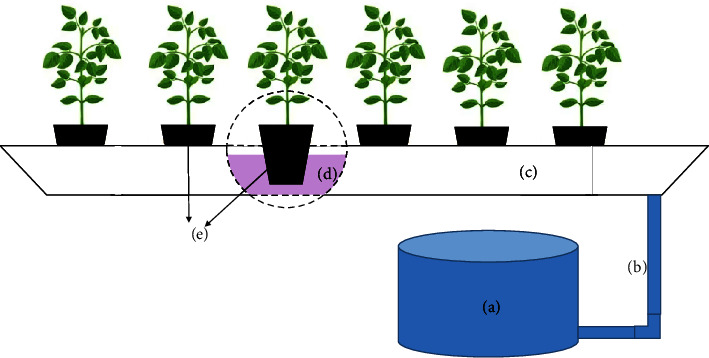
Diagram of the ebb and flow hydroponics system, consisting of a reservoir with a submerged pump (a), pipes conducting the nutrient solution for ebb and flow (b), and white metal channels (c) with nutrient solution (d) and black polyethylene pots containing commercial substrate (e).

An ebb and flow hydroponic system was used, consisting of 2.0-meter long, 0.15-meter wide, and 0.05-meter high channels, flooded with nutrient solution three times a day for 15-minute periods, which allows the solution to be moved to the substrate utilizing capillarity. The pots were placed inside the channel with a spacing of 0.25 m between them (Figure 1).

The nutrient solution was made up of iron chelate, potassium nitrate, potassium sulphate, manganese chelate, copper chelate, zinc chelate, boric acid, crystal monoammonium phosphate, magnesium sulphate, sodium molybdate, nickel sulphate, and cobalt sulphate. This solution provides 8% nitrogen, 8% phosphorus, 30% potassium, 3% sulphur, 1% magnesium, 0.14% iron, 0.04% boron, 0.04% manganese, 0.03% copper, 0.019% zinc, 0.009% molybdenum, 0.006% nickel, and 0.002% cobalt. The pH and electrical conductivity of the solution were regularly adjusted so that they remained at 6.0 ± 0.5 and 1.5 ± 0.3 mS·cm^−1^, respectively.

Five soybean seeds were placed in each pot at a depth of 2 cm. Thinning was conducted 20 days after sowing, when the plants were at the V2 phenological stage, leaving just two plants per pot. The vitamins were then applied by foliar spraying, using a manual sprayer pump, in a volume of 2 mL per pot.

### 2.4. Gas Exchange and Chlorophyll Assessments

Sixty days after sowing, the characteristics of CO_2_ assimilation rate (A), stomatal conductance (gs), intracellular CO_2_ concentration (Ci), and transpiration (E) were assessed during the morning (between 8 a.m. and 10 a.m.) when the plants are in full gas exchange activity. A portable infrared gas analyzer (LCi, ADC Bioscientific, Hertfordshire, United Kingdom) was used on an expanded leaf from the top portion of the plant, and the measurements were performed with parameters of a narrow-leaf cuvette (6.25 cm^2^) at a flow rate of 200 *µ*mol·s^−1^, CO_2_ concentration of 440 ppm, and no light supplementation, after which water use efficiency (A/E) and instantaneous carboxylation efficiency (A/Ci) were calculated. Relative chlorophyll levels were measured using a portable chlorophyll meter (CCM-200, Opti-Sciences, Hudson, USA) on the same leaf as the gas exchange measurements.

### 2.5. Growth and Production Evaluations

To assess the effect of the treatments applied on the growth and development of the crop, the morphological characteristics, plant height, stem diameter, and number of leaves were evaluated 60 days after sowing. The plants were measured using a tape measure from the base of the stem, above the substrate, to the apex to determine height. The diameter was measured using a digital caliper, and the leaves were measured by counting the fully developed trifoliate leaves on the plant.

The soybean crop was grown until it reached the production stage, with the plants being collected 100 days after sowing. The plants were harvested and dried on benches in the growing room. The number of pods per plant, number of grains per pod, number of grains per plant, 100-grain weight, and grain yield per plant were evaluated. The number of grains and pods was determined by manual counting, while the 100-grain weight was determined after counting the grains and weighing them on a precision analytical scale, and the grain yield was obtained by weighing the grains from each plant separately.

### 2.6. Statistical Analysis

The data were submitted to analysis of variance (*F*-test), and the differences between the treatments for inoculation and vitamins were determined using the *t*-test (LSD) and Tukey's test, respectively, at 5% probability. The analyses were conducted using the Sisvar statistical software [19].

Pearson correlation was conducted using the network of correlations between the study variables. Positive correlations were highlighted in green, and negative correlations in red. Determining the thickness of the lines followed a cut-off value of 0.7, corresponding to 70% reliability, so only correlations with averages above this value were highlighted. We also determined the canonical variables between the agronomic and physiological parameters of the soybean crop according to the inoculation and vitamin application treatments. All the analyses were conducted using the R software version (v.4.0.3), using the Qgraph and Candisc packages [20].

## 3. Results

The interaction between vitamins and inoculation influenced the intracellular CO_2_ content (Ci) and stomatal conductance (gs). There were also significant responses to the application of vitamins for the transpiration (E) and water use efficiency (WUE), while inoculation significantly affected the CO_2_ assimilation rate (An), the carboxylation efficiency (CIEI), and the relative chlorophyll index (RCI) (Table 1).

**Figure 2:**
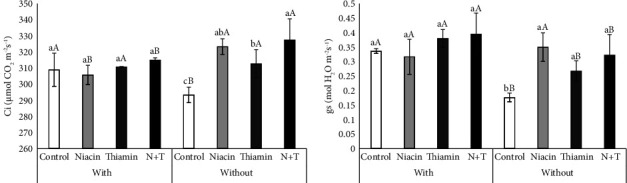
Intracellular CO_2_ concentration (a) and stomatal conductance (b) of soybean plants subjected to different inoculation treatments and vitamin applications. Equal lowercase letters for the vitamin treatments and uppercase letters for inoculation do not differ by the Tukey's and LSD tests at 5% probability, respectively (*n* = 5).

The Ci was not affected by the application of vitamins when combined with inoculation. However, without inoculation, all the vitamins resulted in higher Ci. The absence of inoculation also resulted in higher values for this variable when the plants were treated with niacin and niacin + thiamine (N + T), while plants not treated with vitamins had higher Ci due to inoculation (Figure 2(a)).

For gs, it was found that the application of vitamins affected the characteristic only in noninoculated plants, for which there was a significant increase when treated with vitamin solutions. In addition, inoculation resulted in superior gs for the control, thiamine, and N + T treatments, with no significant change in plants treated with niacin (Figure 2(b)).

The vitamin treatments only affected the E and WUE characteristics, for which the application of niacin stood out with a significant increase of 21.66% and a reduction of 14.09%, respectively, compared to the control treatment. However, inoculation with *Bradyrhizobium japonicum* significantly increased the characteristics of E, An, CIEI, and RCI by around 20.52%, 18.92%, 20.00%, and 46.25%, respectively (Table 2).

For the characteristics relating to growth and yield components, there was an influence of the interaction between the application of vitamins and inoculation with *Bradyrhizobium japonicum* on plant height, number of leaves, number of pods per plant, number of grains per plant, 100-grain weight, and grain yield per plant. In addition, the factors in isolation affected the stem diameter, while the number of grains per pod was unaffected (Table 3).

**Figure 3:**
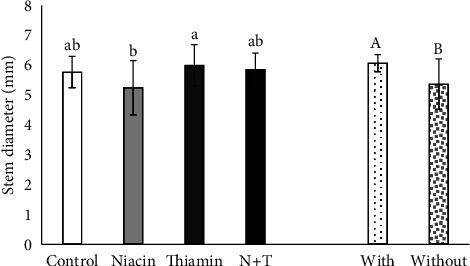
Stem diameter of soybean plants subjected to different inoculation treatments and application of vitamins. Equal lowercase letters for the vitamin treatment and uppercase letters for inoculation do not differ by the Tukey's and LSD tests at 5% probability, respectively (*n* = 5).

For SD, thiamine treatment was superior to niacin treatment, with no significant difference between the control and N + T treatments. It was also found that plants from inoculated seeds had a 13.12% increase in SD compared to noninoculated plants (Figure 3).

**Figure 4:**
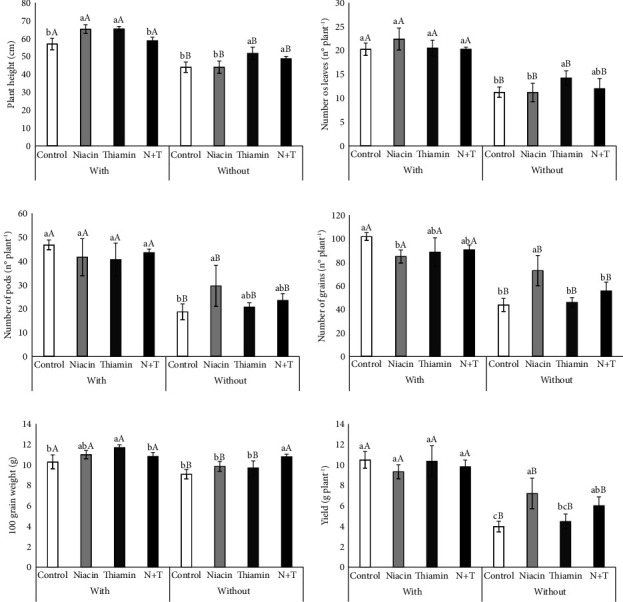
Plant height (a), number of leaves (b), number of pods per plant (c), number of grains per pod (d), 100-grain weight (e), and grain yield (f) of soybean plants subjected to different inoculation treatments and vitamin application. Equal lowercase letters for vitamin treatment and uppercase letters for inoculation do not differ by the Tukey's and LSD tests at 5% probability, respectively.

Applying niacin and thiamin to inoculated plants and thiamin and N + T to noninoculated plants increased PH, while inoculation increased this characteristic regardless of the treatment with the foliar solutions (Figure 4(a)). There were no significant differences between the application of vitamins combined with inoculation for the number of leaves and number of pods per plant, but the application of thiamine and niacin, respectively, resulted in superiority compared to the control treatment. For both characteristics, inoculated plants were superior to noninoculated plants, regardless of the application of foliar solution (Figures 4(b) and 4(c)).

The number of grains per plant was negatively affected by the application of niacin when combined with inoculation, and the presence of *B. japonicum* increased this characteristic. However, when applied to noninoculated plants, niacin was superior to the other treatments, outperforming the control treatment by 66.57% in this condition (Figure 4(d)).

For 100-grain weight and grain yield, inoculation also resulted in superiority compared to untreated plants, except for the N + T treatment for 100-grain weight, which resulted in similarity to the same treatment combined with inoculation when used on noninoculated plants. Among the inoculated plants, thiamine application was superior to the control treatment by 13.52% for 100-grain weight, and there was no difference between the foliar treatments for grain yield in this condition. In contrast, for noninoculated plants, the application of N + T resulted in 100-grain weight being superior, with an increase of 18.95% over the control treatment, while grain yield was increased by 81.43% and 51.31% by the application of niacin and N + T, respectively (Figures 4(e) and 4(f)).

**Figure 5:**
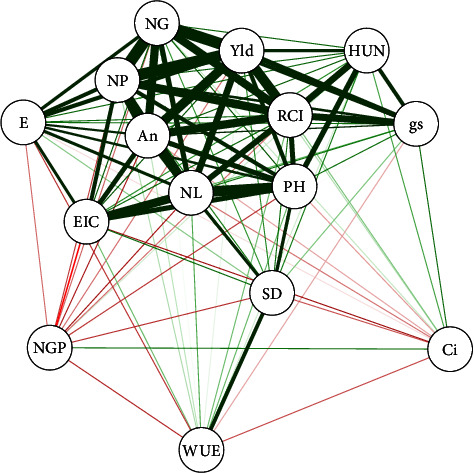
Pearson correlation network among growth variables: plant height (PH), number of leaves (NL), stem diameter (SD); photosynthesis variables: water use efficiency (WUE), relative chlorophyll index (RCI), transpiration (E), intracellular CO_2_ concentration (Ci), stomatal conductance (gs), and CO_2_ assimilation rate (An); grain yield and its components: number of grains per plant (NGP), number of pods per plant (NP), 100-grain weight (HUN), and grain yield per plant (Yld) according to the application of vitamins and seed inoculation. Positive correlations were highlighted in green, while negative correlations were highlighted in red, determining the line thickness followed a cut-off value of 0.7, corresponding to 70% reliability.

The variables in this study generally have a high positive correlation between the attributes analyzed, represented by thick green lines (Figure 5). For the grain yield, which is the most sought after in annual grain crops, there was a significant positive correlation with most of the variables, both in terms of growth and yield components and gas exchange. The CI and number of grains per pod did not correlate with any variable (Figure 5).

**Figure 6:**
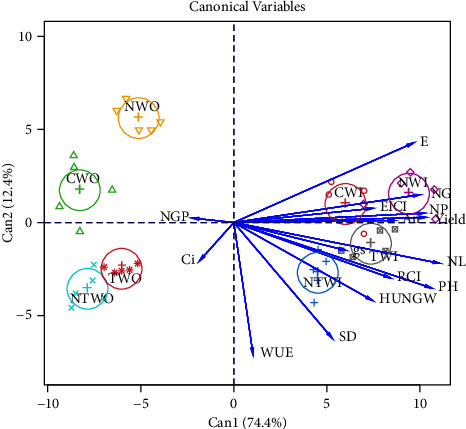
Analysis of canonical variables among growth variables: plant height (PH), number of leaves (NL), stem diameter (SD); photosynthesis variables: water use efficiency (WUE), relative chlorophyll index (RCI), transpiration (E), intracellular CO_2_ concentration (Ci), stomatal conductance (gs), and CO_2_ assimilation rate (An); grain yield and its components: number of grains per plant (NGP), number of pods per plant (NP), 100-grain weight (HUN), and grain yield per plant (Yld) according to the application of vitamins and seed inoculation. Treatment s = C (control); N (niacin); T (thiamine), and NT (niacin + thiamine); WO (without inoculation); and WI (with inoculation).

The canonical variables showed that all the treatments with foliar solution (control, niacin, thiamine, and N + T) were more effective in grain yield, growth, and gas exchange when combined with inoculation with *Bradyrhizobium japonicum*. This trend was not seen only for the number of grains per pod and Ci (Figure 6).

## 4. Discussion

Our findings revealed that the most significant positive effects on the physiological, growth, and yield parameters of soybeans in a hydroponic cultivation system were related to inoculation with *Bradyrhizobium japonicum* (Figure 6). In this sense, the increase in gas exchange capacity and relative chlorophyll levels are related to the changes triggered by the association between the bacteria and the plants.

Biological nitrogen fixation is one of the main effects triggered by the *Bradyrhizobium japonicum* species and is essential when it comes to meeting the nitrogen demand of soybean plants. According to Nogueira et al. [21], the use of strains of bacteria from the *Bradyrhizobium* genus promotes the effect of symbiosis between the plant and the microorganisms, which can nodulate the roots, from which they take their food and protection and, as a means of exchange, provide the plant with nutrients. Bacteria capture atmospheric N through the action of the nitrogenase enzyme and convert it into forms that the plant can assimilate.

In addition to nitrogen, the presence of *B. japonicum* promotes the production of hormones related to growth, such as IAA, which stimulates root development and the absorption of nutrients by plants. This set of characteristics triggers a greater capacity for gas exchange, increases the production of photosynthetic pigments (Table 2), and makes it possible to increase vegetative and reproductive development (Figures 3 and 4), verified in our study and agreement with other works [10, 11, 22] since these characteristics are mostly positively correlated (Figure 5).

The effects promoted by inoculation are significant to the point of overshadowing the effects of vitamin application. However, in the absence of *B. japonicum*, both niacin and thiamine, applied alone or combined, resulted in better gas exchange conditions, as seen for gs and E (Figure 1(b) and Table 2), which have a positive correlation with yield components such as the number of pods per plant, number of grains per pod, 100-grain weight, and grain yield (Figure 5). In this sense, niacin stands out since its application increases reproductive structures and yield, even when combined with thiamine (Figure 4). It is also possible to see that the positive interactions between the inoculation and the application of vitamins have an additive (Figure 4), synergy character, due to the different forms of positive action on the characteristics of the plant, which can occur through numerous metabolic pathways, however without harming its development.

Vitamin B3 is essential for plants since it participates in the constitution of NAD and NADH, acting directly in the transfer of hydrogen ions in energy-releasing reactions [15], affecting the assimilation of nitrogen by plants since this coenzyme actively participates in the reduction of nitrate into ammonia [23]. When applied exogenously, this vitamin also has a direct influence on greater photosynthetic capacity and the accumulation of mass in the aerial organs [4], which may be related to the gain in height development when it was applied to inoculated plants (Figure 4(a)).

For leguminous species, it has been found that the application of vitamin B3 significantly increases the growth and yield of fava bean grains, which is related to the increase in the levels of carbohydrates, free amino acids, proline, and auxin in the plant tissues [24]. Similar effects were obtained for the pea crop, for which increases in reserves, hormone, and leaf pigment resulted in gains in growth and yield [25], and for the bean plant, for which there was an increase in plant vigor during initial growth [17].

The positive effects of the application of thiamine, either alone or combined with niacin, on plant height, number of pods per plant, 100WG, and grain yield (Figures 4(a), 4(b), 4(e), and 4(f)) are stimulated, according to Kaya et al. [26], due to the exogenous application interfering with tissue protection through antioxidant action, which may have favored higher CO_2_ concentration and stomatal conductance (Figures 1(a) and 1(b)), since this antioxidant character enables the proper functioning of photosynthetic processes, which are essential for obtaining energy for vegetative and reproductive growth [23]. In addition, when studying the exogenous application of thiamine and niacin to sugarcane, Ramos et al. [27] also confirmed the positive effect of applying thiamine alone at a concentration of 0.1 g·L^−1^ or combined with niacin at a concentration of 50 mg·L^−1^, as the vitamins contributed to better functioning of the photosynthetic apparatus and stomatal morphology.

According to Rodrigues et al. [28], the use of molecules such as thiamine in soybean crops during the grain-filling phase has produced positive results, given that thiamine is a cofactor for various enzymes that are used in the metabolic processes of carbohydrates and amino acids, providing more energy for plant metabolism. Positive results with foliar application of thiamine were also presented by Al-Hayani and Al-Jumaili [29] with mung beans (*Vigna radiata* L.), for which the application of 450 mg·L^−1^ promoted greater development of leaf area, pod production per plant, and grain yield, which were associated with the action of thiamine on meristematic cells and as a cofactor in the Krebs cycle, affecting the activation and regulation of plant growth.

Our results, together with the available information, show that both technologies have the potential to be applied to soybeans grown in hydroponic systems. However, the innovative nature of our findings means that new studies should be conducted to determine management methods that allow the maximum expression of the effects of both inoculation and the application of vitamins, separately or combined so that technical recommendations can be made for commercial crops.

## 5. Conclusions

Inoculation with *Bradyrhizobium japonicum* in soybean seeds grown in a hydroponic system significantly benefits the development and production of grains, mainly when combined with vitamin solutions at the V2 stage of the soybean plants.

The application of vitamins, especially niacin, has the potential to be used alone or combined with thiamine in hydroponic soybeans that are not inoculated or inoculated, respectively, with *Bradyrhizobium japonicum*.

The interaction between inoculation and foliar application of vitamins can be used in hydroponic soybean cultivation.

## Figures and Tables

**Table 1:** Summary of the analysis of variance for internal CO_2_ concentration (Ci), transpiration rate (E), net photosynthetic rate (An), stomatal conductance (gs), water use efficiency (WUE), carboxylation efficiency (CIEI), and relative chlorophyll index (RCI) of soybean plants subjected to different inoculation managements and applications of vitamins.

SV	DF	*F* values			

		Ci	E	An	gs

Block	4	0.23	0.47	0.69	1.00
Vitamin (V)	3	11.50^*∗∗*^	32.90^*∗∗*^	0.50^ns^	7.85^*∗∗*^
Inoculation (I)	1	2.77^ns^	126.53^*∗∗*^	44.86^*∗∗*^	26.03^*∗∗*^
V × I	3	8.89^*∗∗*^	1.07^ns^	1.93^ns^	7.04^*∗*^
Error	28				

CV (%)		2.50	5.27	8.14	15.26
Mean		312.16	2.95	11.92	0.32

SV	DF				

		WUE	CIEI	RCI	

Block	4	0.48	1.03	0.19	
Vitamin (V)	3	9.46^*∗∗*^	0.86^ns^	2.54^ns^	
Inoculation (I)	1	3.28^ns^	23.88^*∗∗*^	53.33^*∗∗*^	
V × I	3	2.82^ns^	1.28^ns^	2.25^ns^	
Error	28				

CV (%)		7.51	12.23	16.25	
Mean		4.15	0.039	12.65	

^*∗*^significant at 5% probability; ^*∗∗*^significant at 1% probability; ns = not significant; CV = coefficient of variation; DF = degrees of freedom.

**Table 2:** Transpiration (E), assimilation rate (A), water use efficiency (WUE), carboxylation efficiency (CIEI), and relative chlorophyll index (RCI) of soybean plants subjected to different inoculation management and application of vitamins.

Vitamin	E (mmol H_2_O m^−2^·s^−1^)	An (*µ*mol CO_2_ m^−2^·s^−1^)	WUE (*μ*mol CO_2_ mmol H_2_O^−1^)	CIEI (*µ*mol CO_2_ m^−2^·s^−1^)	RCI (−)
Control	2.77 ± 0.32 b	11.75 ± 1.34 a	4.33 ± 0.18 a	0.040 ± 0.004 a	11.55 ± 3.69 a
Niacin (N)	3.37 ± 0.36 a	11.97 ± 1.05 a	3.72 ± 0.44 b	0.040 ± 0.008 a	12.07 ± 2.66 a
Thiamin (T)	2.91 ± 0.37 b	12.20 ± 1.53 a	4.16 ± 0.28 a	0.038 ± 0.005 a	13.10 ± 3.80 a
T + N	2.77 ± 0.25 b	11.76 ± 1.81 a	4.40 ± 0.38 a	0.037 ± 0.006 a	13.86 ± 2.57 a

*Inoculation*					
With	3.23 ± 0.29 a	12.95 ± 0.96 a	4.24 ± 0.30 a	0.042 ± 0.005 a	15.02 ± 2.60 a
Without	2.68 ± 0.29 b	10.89 ± 0.99 b	4.06 ± 0.50 a	0.035 ± 0.004 b	10.27 ± 1.68 b

Lowercase letters in the column for each variable do not differ (Tukey's and LSD) at 5% probability (*n* = 5).

**Table 3:** Summary of the analysis of variance (ANOVA) for plant height (PH), number of leaves (NL), stem diameter (SD), number of pods per plant (NP), number of grains per plant (NG), number of grains per pod (NGP), 100-grain weight (100 GW), and grain yield of soybean plants subjected to different inoculation managements and vitamin application.

SV	DF	*F* values			

		PH	NL	SD	NP

Block	4	3.68	0.34	0.61	0.68
Vitamin (V)	3	20.78^*∗∗*^	1.77^ns^	3.48^*∗*^	1.51^*∗∗*^
Inoculation (I)	1	396.68^*∗∗*^	257.00^*∗∗*^	16.02^*∗∗*^	144.74^*∗∗*^
V × I	3	10.69^*∗∗*^	3.57^*∗*^	2.72^ns^	3.90^*∗*^
Error	28				

CV (%)		4.23	10.36	9.73	15.86
Mean		54.36	16.51	5.71	33.14

SV	DF				

		NG	NGP	100 GW	Yield

Block	4	0.17	0.43	1.06	0.41
Vitamin (V)	3	3.37^*∗*^	0.02^ns^	11.23^*∗∗*^	2.18^ns^
Inoculation (I)	1	203.01^*∗∗*^	1.67^ns^	52.34^*∗∗*^	204.38^*∗∗*^
V × I	3	13.70^*∗∗*^	1.86^ns^	7.07^*∗∗*^	9.74^*∗∗*^
Error	28				

CV (%)		11.24	5.53	4.59	13.17
Mean		73.00	2.26	10.40	7.71

^*∗*^significant at 5% probability; ^*∗∗*^significant at 1% probability; ns = not significant; CV = coefficient of variation; DF = degrees of freedom.

## Data Availability

The data used in this study are available from the author upon request.
